# Stress Across Generations: DNA Methylation as a Potential Mechanism Underlying Intergenerational Effects of Stress in Both Post-traumatic Stress Disorder and Pre-clinical Predator Stress Rodent Models

**DOI:** 10.3389/fnbeh.2019.00113

**Published:** 2019-05-28

**Authors:** Sriya Bhattacharya, Audrey Fontaine, Phillip E. MacCallum, James Drover, Jacqueline Blundell

**Affiliations:** ^1^Department of Psychology, Memorial University of Newfoundland, St. John’s, NL, Canada; ^2^ Institut des Systèmes Intelligents et de Robotique (ISIR), Université Pierre et Marie Curie, Sorbonne Universités, Paris, France

**Keywords:** PTSD, predator stress, intergenerational, DNA methylation, glucocorticoid receptors, FKBP5

## Abstract

Although most humans will experience some type of traumatic event in their lifetime only a small set of individuals will go on to develop post-traumatic stress disorder (PTSD). Differences in sex, age, trauma type, and comorbidity, along with many other elements, contribute to the heterogenous manifestation of this disorder. Nonetheless, aberrant hypothalamus-pituitary-adrenal (HPA) axis activity, especially in terms of cortisol and glucocorticoid receptor (GR) alterations, has been postulated as a tenable factor in the etiology and pathophysiology of PTSD. Moreover, emerging data suggests that the harmful effects of traumatic stress to the HPA axis in PTSD can also propagate into future generations, making offspring more prone to psychopathologies. Predator stress models provide an ethical and ethologically relevant way to investigate tentative mechanisms that are thought to underlie this phenomenon. In this review article, we discuss findings from human and laboratory predator stress studies that suggest changes to DNA methylation germane to GRs may underlie the generational effects of trauma transmission. Understanding mechanisms that promote stress-induced psychopathology will represent a major advance in the field and may lead to novel treatments for such devastating, and often treatment-resistant trauma and stress-disorders.

## Post-Traumatic Stress Disorder (PTSD)

Individuals exposed to highly traumatic experiences (e.g., physical assault, rape, natural disaster, kidnapping, combat, etc.) can develop post-traumatic stress disorder (PTSD). The DSM-V classifies PTSD as a stress and trauma-related disorder, defined by a set of symptom clusters that appear for at least 30 days following severe trauma. Symptoms include *re-experiencing* of the trauma (unwanted intrusion of the memory in the form of thoughts, nightmares, and flashbacks, intense upset evoked by conditioned cues or environmental stimuli), *avoidance* of cues related to the traumatic event (situations, places, activities), and *hyperarousal* (increased startle response, irritability, sleep problems). Negative disturbances in mood and cognition (dissociative amnesia, negative affect, and anhedonia) are also recognized as core symptoms (American Psychiatric Association, 2013). Women are twice as likely to develop PTSD as men, and the disorder is often comorbid with other anxiety disorders, as well as depression and substance abuse (Kessler et al., 1995). Not only can exposure to trauma affect the psychological status of an individual, but it can also have deleterious consequences on the individual’s social, professional, and family life. As a result, the fallout from trauma carries a large burden for individuals and society (Kessler et al., 1995).

Epidemiological studies have found that between 37% and 92% of people report past exposure to at least one traumatic event (Van Ameringen et al., 2008; Kilpatrick et al., 2013; Atwoli et al., 2015; Benjet et al., 2016; Kessler et al., 2017). Of those, between 25% and 35% of trauma survivors go on to develop PTSD (Yehuda, 2001; Kessler et al., 2005). These figures contribute to the lifetime prevalence of the disorder which is currently estimated at 6.1% in the United States and 9.2% in Canada (Van Ameringen et al., 2008; Goldstein et al., 2016). PTSD is, therefore, one of the most common psychiatric disorders as most anxiety and affective related disorders [e.g., generalized anxiety disorder (GAD), agoraphobia, panic attacks, obsessive compulsive disorder (OCD), major depressive disorder, bipolar disorder] for comparison have relatively lower lifetime prevalence, ranging from 0.7% to 5% (Kessler et al., 2012; Bandelow and Michaelis, 2015; Statistics Canada, 2015). Among “highly exposed” groups (e.g., low-income, low social support, urban populations), lifetime rates of PTSD can be as high as 40% (Breslau et al., 1998). In the wake of large-scale disastrous events, incidence and prevalence of PTSD to populations endemic to the area involved can often by shifted sharply upward, like those affected in the New York City area or the Mississippi Delta following the 9/11/2001 terrorist attacks or Hurricane Katrina in 2005, respectively (Galea et al., 2002, 2008). Nonetheless, trauma event type confers differences in risk for PTSD. Direct interpersonal victimization related to rape, physical abuse, and kidnapping has the highest conditional risk, while other trauma event categories such as automobile accident or natural disaster are at low risk for PTSD prevalence (Kilpatrick et al., 2013; Benjet et al., 2016; Kessler et al., 2017). In addition, a dose-response relationship exists between symptom severity and frequency of trauma experience: the more traumatic events a person experiences, the greater the intensity of their PTSD symptoms (Binder et al., 2008).

Although many individuals who present with the symptoms of PTSD actively seek out psychotherapy, psychopharmacology, or both (Van Ameringen et al., 2008) only about 60% are responsive to interventions (Önder et al., 2006), with only about a third of patients achieving full remission (Berger et al., 2009). The disparity in those achieving full remission, coupled with epidemiological studies estimating the lifetime prevalence of PTSD at 5%–10% for the general population (Van Ameringen et al., 2008), suggests a desperate need for understanding the mechanisms that contribute to the vulnerability, development, and maintenance of PTSD ultimately leading to the identification of candidate treatments (Reul and Nutt, 2008; Hauger et al., 2012; Steckler and Risbrough, 2012).

As noted above, traumatic stress can have deleterious effects on an individual’s mental health. However, recent data suggests that the harmful effects of traumatic stress during one’s lifetime can propagate into future generations (Blaze and Roth, 2015), perhaps leading to an increased vulnerability to the development of psychopathology. This review will examine recent work on the intergenerational effects of stress in both human (with PTSD) and rodent (predator stress) studies, with a focus on DNA methylation as a potential underlying mechanism. As predator stress is ecologically relevant and considered one of the most comprehensive animal models of PTSD (see Deslauriers et al., 2017), our discussion will focus on research using this model. For the purposes of this review, we define intergenerational effects of stress similar to that described by Klengel et al. (2015). Briefly, intergenerational transmission involves direct stress exposure to the parental (F0) generation and subsequent offspring generation (F1) by means of either the developing germ cell or fetus. If the stress exposure occurred while the fetus (F1) was developing *in utero*, intergenerational transmission occurs in the F2 generation. This is in contrast to transgenerational whereby the germ cells have *not* been exposed to stress.

## Human Studies: Stress Effects Across Generations

There is a variety of research showing intergenerational effects of stress. In a landmark study by Solomon et al. (1988), Israeli veterans of the 1982 Lebanon War who were offspring of Holocaust survivors were more likely to develop PTSD than other Israeli soldiers following their military experiences who did not have parents interned in Nazi concentration camps (Solomon et al., 1988). Likewise, PTSD in former Israeli soldiers captured and held as prisoners of war during the 1973 Yom Kippur War positively correlated with offspring PTSD (Zerach et al., 2017). Furthermore, in a study of Cambodian refugees living in the United States, parental PTSD predicted higher rates of child PTSD for older children. A gradient effect was found such that when neither parent had PTSD, 12.9% of the children had PTSD; when one parent had PTSD, 23.3% of the children had PTSD; and when both parents had PTSD, the percentage of children with PTSD jumped to 41.2% (Sack et al., 1995). In a more recent report, offspring of parents suffering from PTSD that survived the 1994 Tutsi genocide in Rwanda showed greater secondary traumatization symptoms and were less resilient (Shrira et al., 2019). In addition to intergenerational effects, data suggests transgenerational effects of stress. For example, offspring of Israeli Holocaust survivors had a higher incidence of PTSD, mood disorders, anxiety disorders, and substance abuse disorders three generations downstream (Barocas and Barocas, 1979; Dasberg, 1987; Scharf, 2007; Yehuda et al., 2008, 2015). Overall, these data suggest that parental traumatic stress make offspring more vulnerable to mental illness.

### Stress Hormones

Stress hormones appear to play a key role in the development of, and vulnerability to, PTSD. Cortisol (principal human stress hormone; corticosterone is the main glucocorticoid in most other species) coordinates and prepares the body to respond to environmental demands and stressors to achieve systems homeostasis. Selye (1956) was the first to demonstrate a common pathway of physiological activity in response to stress. This pathway was later dubbed the hypothalamic-pituitary-adrenal (HPA) axis. During a stressful event, cells of the paraventricular nucleus of the hypothalamus respond by secreting corticotropin-releasing hormone (CRH) into capillaries in the median eminence of the hypothalamus. CRH released into this portal capillary system stimulates neurosecretory cells in the anterior pituitary which in turn release adrenocorticotropin hormone (ACTH). From there, ACTH travels through the blood stream and acts on the cortex of the adrenal gland where it stimulates secretory cells to release glucocorticoids, particularly cortisol, into the general circulatory system. Cortisol prepares the body to adapt to current stressors by suppressing immune system activity, counteracting insulin, supporting increased glucose availability (e.g., gluconeogenesis), and regulating water retention and electrolytic balance in the kidneys (Khani and Tayek, 2001; Dunlop and Wong, 2019). Cortisol also acts to decrease the activity of paraventricular nucleus and anterior pituitary, negatively influencing its own release. This regulator mechanism limits the stress response helping return the body to homeostasis and is often referred to as the negative feedback loop (Sapolsky et al., 1985). As the term stress is ambiguous and not well defined, it is important to note that various daily events that many might interpret as innocuous or pleasurable, such as exercise or sex, evoke this canonical HPA axis stress response (Stranahan et al., 2008; Jokinen et al., 2017; Finke et al., 2018). Nevertheless, unlike sex and exercise, the stressors that satisfy Criteria A in the DSM-5, while quite varied, are traumatic, sudden, unexpected, involuntary, and uncontrollable (American Psychiatric Association, 2013; Kilpatrick et al., 2013; Kessler et al., 2017). Dysregulated or aberrant HPA axis activity, especially in terms of cortisol, is often postulated as part of the etiology and pathophysiology of PTSD in response to traumatic events. The direction of this altered neuroendocrine activity that might confer a susceptibility to developing and maintaining PTSD, however, has yielded inconsistent findings (Zoladz and Diamond, 2013).

Mason et al. (1986) were the first to report lower mean 24-h basal urinary cortisol levels from inpatient PTSD combat veterans. Although this study only compared different psychiatric groups, subsequent studies have shown similar low cortisol levels relative to healthy controls (Yehuda et al., 1993; Kanter et al., 2001; Rohleder et al., 2004). Conversely, other studies have reported increased basal cortisol levels (Lemieux and Coe, 1995; Carrion et al., 2002; Young and Breslau, 2004) or no difference in individuals with PTSD compared to healthy controls (Duval et al., 2004; Yehuda et al., 2004; Otte et al., 2007). Discrepancies in reported cortisol levels concern issues around, but not limited to, sex, length of combat exposure in veterans, differences in trauma type, childhood trauma, current PTSD status, differences in naturally fluctuating levels of cortisol throughout the day (at awakening or peak, nadir, fastening state), comorbidity with other psychiatric disorders such as major depressive disorder and substance use disorders, plasma vs. urinary vs. cerebral spinal fluid cortisol levels, and lack of statistical power (Zoladz and Diamond, 2013; Dunlop and Wong, 2019). Given such inconsistences, these findings suggest that basal cortisol abnormalities might only represent a subset of the manifestation of PTSD. As such, basal cortisol is a rather dubious biomarker for PTSD. Zoladz and Diamond (2013), suggest investigating a more comprehensive role for cortisol in multiple physiological processes. In particular, it is suggested that impaired HPA axis functioning diminishes negative feedback and increases sympathetic nervous system and immune system activity, both linked to PTSD etiology and pathophysiology (Zoladz and Diamond, 2013).

Similar to the heterogeneous findings of baseline cortisol activity, mixed evidence linking altered cortisol levels in the immediate aftermath of an acute traumatic experience to PTSD have been reported. Specifically, one study found a negative correlation between diminished cortisol levels peri-trauma and to the development of PTSD (Ehring et al., 2008), whereas other studies have found no correlation (Resnick et al., 1995; Heinrichs et al., 2005), or a positive correlation, especially in relation to childhood trauma and the development of PTSD months later (Lipschitz et al., 2003; Delahanty et al., 2005; Pfeffer et al., 2007). Here, it is probable that the ontogeny of HPA axis during childhood contributes differently to the development of PTSD compared to maladaptive adulthood responses. In adults, administration of hydrocortisone to patients undergoing very invasive surgeries show fewer PTSD symptoms in follow-up sessions (Schelling et al., 2004, 2006), providing credence for perturbed cortisol functioning in adults’ response to acute traumatic stress that goes on to develop PTSD. Moreover, studies investigating cortisol levels in the dexamethasone suppression test and trier social stress test of individuals with PTSD have shown rather consistently robust inhibition of cortisol in response to synthetic glucocorticoids or psychosocial stress (Yehuda, 2002; Zoladz and Diamond, 2013; Wichmann et al., 2017).

Interestingly, alterations in stress hormone release have also been observed in the offspring of individuals with PTSD. Yehuda et al. (2000) found lower serum cortisol levels and greater cortisol suppression in the offspring of Holocaust survivor’s suffering from PTSD compared to healthy subjects who were not offspring of Holocaust survivors (Yehuda et al., 2000). Further, pregnant women who developed PTSD in response to the September 11, 2001, World Trade Center collapse had infants with lower cortisol levels compared with infants of mothers who did not develop PTSD (Yehuda et al., 2005). Similarly, Yahyavi et al. (2015) found that offspring of Iranian combat veterans from the Iran-Iraq war with a current or past history of PTSD had significantly lower serum cortisol levels than offspring of combat veterans without a history of PTSD. Moreover, beyond diurnal changes in stress hormones, youth offspring of mothers with PTSD also show a blunted, maladaptive, cortisol saliva response to an acute laboratory stressor (Danielsona et al., 2015), a response seen likewise in female PTSD patients (Pierrehumbert et al., 2009; Zaba et al., 2015; Wichmann et al., 2017).

## Epigenetic Modifications: DNA Methylation and PTSD

Epigenetic modifications have been espoused to help explain the transmission of experience to future generations. Epigenetic modifications are changes in the transcriptional potential of a cell by the environment, which occur independent of alterations in the gene sequence. Epigenetic modifications provide a mechanism that links genes and environment while playing an important role in the modulation of behavioral responses to stress (Day and Sweatt, 2011). Although the mechanism by which epigenetic modifications lead to the transmission of environmental influences to subsequent generations is not known, one possibility involves the transmission of DNA methylation (Meaney, 2001; Day and Sweatt, 2011; Dias and Ressler, 2014).

DNA methylation is one of the most broadly studied and well-characterized epigenetic modifications. DNA methylation plays a significant role in the maintenance of cellular identity and heritable changes in gene expression throughout the cell cycle, typically by prohibiting DNA transcription by the addition of a methyl group to a gene promoter region. Indeed, DNA methylation may be important in fundamental mechanisms for the induction and stabilization of PTSD (Zovkic et al., 2013).

As can be seen in Table 1, several studies have identified alterations in methylation states of specific genes in people with PTSD. Of the gene methylation studies in people with PTSD, the most commonly studied target is the glucocorticoid receptor (GR), a major regulator of the HPA-axis. GR, also known as Nr3c1 (nuclear receptor subfamily 3, group C, member 1), is the receptor to which cortisol and other glucocorticoids bind. The GR is expressed in almost every cell in the body and regulates genes controlling the development, metabolism, and immune response. In the absence of cortisol or corticosterone hormone, GR resides in the cytosol complexed with a variety of proteins including heat shock protein 70 and 90 (hsp70 and 90), and the protein chaperone FK506-binding protein (Fkbp5). Labonté et al. (2014) found that individuals with lifetime PTSD had lower morning cortisol release, higher mRNA expression of the human GR (hGRtotal, 1B, and 1C) and lower overall methylation levels in hGR 1B and 1C promoter regions. Similarly, lower *Nr3c1* 1F promoter methylation was observed in combat veterans with PTSD compared with combat veterans without PTSD (Yehuda et al., 2014). Interestingly, DNA methylation of the Nr3c1 promotor appears to be sex-specific as methylation at the *Nr3c1* 1F promoter was linked to traumatic memories and PTSD risk in male, but not in female genocide survivors in Rwanda (Vukojevic et al., 2014). However, much more research is necessary to fully assess the role of sex on DNA methylation of specific targets in people with PTSD.

**Table 1:** Selective human studies supporting the role of DNA methylation in post-traumatic stress disorder (PTSD).

Reference	Candidate gene	Epigenetic changes	Sample size	Generation study carried out through
Koenen et al. (2011)	*Slc6a4*	Lower *Slc6a4* methylation levels in blood tissue and higher number of traumatic events increased risk for PTSD	100	F0
Ressler et al. (2011)	*Adcyap1r1*	*Adcyap1r1* methylation levels were observed in the peripheral blood of PTSD subjects compared with control	94	F0
Rusiecki et al. (2013)	*Il8, Il16, Il18*	Increased serum *Il18* methylation in combat veterans who developed PTSD, but decreased *I/18* methylation levels in veterans without PTSD	150	F0
Norrholm et al. (2013)	*Comt*	Higher blood level methylation of *Comt* are associated with impaired fear inhibition	270	F0
Yehuda et al. (2013)	*Nr3c1, Fkfbp5*	Pre psychotherapy* Nr3c1* 1F promoter methylation in blood tissue positively correlated with improvements in symptoms, while decreased *Fkbp5* methylation occurred concomitantly with recovery from PTSD	16	F0
Labonté et al. (2014)	*Nr3c1*	Increased *Nr3c1*mRNA expression and decreased overall *Nr3c1* 1B and 1C promoter methylation levels in individuals blood with lifetime PTSD	46	F0
Vukojevic et al. (2014)	*Nr3c1*	In the peripheral blood, methylation of *Nr3c1* 1F promoter is linked to traumatic memories and PTSD risk in male survivors of the Rwandan genocide	152	F0
Yehuda et al. (2014)	*Nr3c1*	*Nr3c1* 1F promoter methylation inversely correlated with symptoms severity in the blood tissue of combat veterans with PTSD	122	F0
Yehuda et al. (2014)	*Nr3c1*	Offspring with paternal PTSD showed higher *Nr3c1* 1F promoter methylation in the blood tissue if maternal PTSD was not present. Offspring with maternal and paternal PTSD showed lower methylation	95	F0, F1(Intergeneration)
Perroud et al. (2014)	*Nr3c1, Nr3c2*	Higher plasma levels methylation at promoter 1F for *Nr3c1* in female survivors of genocide and their offspring compared to controls, but no differences observed in *Nr3c2* methylation	25	F0, F1(Intergeneration)
Yehuda et al. (2016)	*Fkbp5*	Holocaust survivors showed increased methylation of the promotor region for *Fkbp5*, while their offspring showed the opposite in their blood tissue	71	F0, F1(Intergeneration)
Kertes et al. (2017)	*Bdnf*	Maternal experiences of war trauma were associated with higher *Bdnf* methylation in umbilical cord blood, placental tissue, and lower methylation in maternal venous blood.	24	F0, F1(Intergeneration)
Serpeloni et al. (2017)	*Barx1, Cftr, Corin, Smyd3*	Grandmaternal exposure to CDV during pregnancy was significantly associated with decreased methylation in *Corin*, *Smyd3*, and *Barx1*, and increased methylation of *Cftr* in grandchildren’s saliva samples	121	F0, F1, F2, F3 (Intergeneration)
Kim et al. (2017)	*Bdnf*	Subjects with PTSD showed a higher methylation *Bdnf* promoter I region in their blood tissue compared with those without PTSD	248	F0
Voisey et al. (2019)	*Bdnf*	Decreased methylation at three *Bdnf* sites were observed in combat exposed PTSD veterans blood compared with control.	96	F0

*Abbreviations: adenylate cyclase-activating polypeptide Type I receptor gene (*Adcyap1r1*), amplification of inter-methylated sites (*Aims*), BARX homeobox 1 (*Barx1*), brain-derived neurotrophic factor (*Bdnf*), catechol-O-methyltransferase gene (*Comt*), community and domestic violence (CDV), corin, serine peptidase genes (Corin), cystic fibrosis transmembrane conductance regulator (*Cftr*), FK506 binding protein 5 (*Fkbp*), nuclear receptor subfamily 3 group C member 1 gene (*Nr3c1*, glucocorticoid receptor gene), insulin like growth factor 2 (*Igf2*), interleukin like chemokines (*Il8, Il16, Il18*), nuclear receptor subfamily 3 group C member 2 gene (*Nr3c2*, mineralcorticoid receptor gene), SET and MYND domain containing 3 (*Smyd3*), solute carrier family 6 member 4 (*Slc6a4*)*.

As described by Klengel et al. (2015), it was once thought that DNA methylation was completely erased during development of the primordial germ cells and during fertilization. However, this erasure, or reprogramming, appears not to be complete. Evidence for certain loci that escape reprogramming is growing with examples including imprinted genes and repetitive elements (Kobayashi et al., 2012; Radford et al., 2014; Tang et al., 2015). Hence, altered GR promoter methylation may be one mechanism by which parental stress is translated into changes in gene expression and physiology, ultimately resulting in psychologically vulnerable offspring phenotypes (see Table 1). Perroud et al. (2011, 2014) examined the impact of the Tutsi genocide on the children of women who were pregnant while genocide was ongoing in Rwanda. In 2011, more than 20% of the Rwandan population met the criteria for PTSD. Peripheral blood leukocytes were obtained and methylation levels of the promoter regions of the GR *NR3C1* was examined in trauma-exposed woman and their children. As expected, both mothers exposed to genocide and their children had significantly higher levels of PTSD than the control group. They also showed higher methylation levels at exon 1F promoter of *Nr3c1*, at CpG3-CpG9. Furthermore, there was a negative correlation between *Nr3c1* methylation and glucocorticoid levels in plasma (Perroud et al., 2011, 2014). Changes in methylation of the GR receptor appears to be dependent on parental status of PTSD. Offspring with just paternal PTSD showed higher GR-1F promoter methylation, whereas offspring with both maternal and paternal PTSD showed lower methylation (Yehuda et al., 2014). Interestingly, in comparison to demographical controls, Holocaust survivors showed increased methylation of the promoter region for FK506 binding protein 5 (FKBP5), a protein that lowers the affinity of cortisol when it is bound to GR; thereby potentially hindering the negative HPA axis feedback loop (Binder, 2009), while methylation at this site was lower in Holocaust survivor offspring (Yehuda et al., 2016). Although gender-specific effects cannot be disentangled in this study, Yehuda et al. (2016) suggest that *Fkbp5* hypermethylation, leading to decreased *Fkbp5* expression and increased GR sensitivity in the F0 mothers, may result in lowered circulating glucocorticoid levels during pregnancy, promoting demethylation in the fetus to optimize or increase glucocorticoid levels. Alternatively, preconception or postnatal social influences may also influence offspring cortisol levels and hence regulate *Fkbp5* methylation levels. Currently, whether changes in glucocorticoids in offspring reflect intergenerational consequences of parental exposure or offspring recalibration of glucocorticoid regulation is not known. Future studies, perhaps using animal models (discussed below), are necessary to fully understand the role of GR methylation in development and vulnerability to mental illness.

While epigenetic studies investigating the intergenerational effects of stress have primarily looked at changes in the methylation state of GR, recent reports suggest that other targets may play a role in this transmission (see Table 1). While methylation of the *Bdnf* gene promoter region has been associated with the development of PTSD (Kim et al., 2017; Voisey et al., 2019), a recent report suggests that maternal trauma exposure may be linked to high *Bdnf* methylation levels in offspring (Kertes et al., 2017). Among 24 mothers and newborns in the eastern Democratic Republic of Congo, a region with extreme conflict and violence to women, maternal experiences of war trauma and chronic stress were associated with higher *Bdnf* methylation in umbilical cord blood, placental tissue, and lower methylation in maternal venous blood. While the studies described above examined parental and first-generation methylation status of specific genes, Serpeloni et al. (2017) examined the grandchildren of grandmothers exposed to psychosocial stress during pregnancy. Grand maternal exposure to community and domestic violence (CDV) during pregnancy was significantly associated with decreased methylation of the *CORIN* (corin, serine peptidase), *SMYD3* (SET and MYND domain-containing protein 3, is a histone methyltransferase), and *BARX1* (BARX Homeobox 1 is a protein-coding gene) genes, as well as increased methylation of the *CFTR* (cystic fibrosis transmembrane conductance regulator) gene in the grandchildren. *CFTR* and *CORIN* genes are involved in circulatory system processes and congenital abnormalities and dysregulation of these genes impact the release of vitamin D, blood pressure regulation, heart failure and hypertension. *SMYD3* and* BARX1* genes are involved in embryonic development, craniofacial development, odontogenesis, and stomach organogenesis. The exact role methylation of these genes plays in the intergenerational effects of stress, however, has not been elucidated.

In all, there appears to be strong support for epigenetic regulation, specifically DNA methylation, in human PTSD (see Table 1). Furthermore, evidence suggests that these epigenetic changes may be passed on to future generations and lead to a vulnerability to psychiatric illnesses in the offspring. Although these findings are exciting, several limitations should be noted. For instances, low sample size, age variability, a lack of replication and sufficient controls, and a paucity of information on sex effects/differences and ancestry information (Rady et al., 2010; Zannas et al., 2016; Lacal and Ventura, 2018) plague these studies. Furthermore, maternal and paternal lineages may have differing effects on the epigenetic transmission of stress. Moreover, the exact nature of the transmission is dependent on which parent underwent the stressor (and when) as the maternal lineage can be observed in the F0, F1 and F2 generations, and the transgenerational phenotype in F3, whereas the paternal lineage can be seen in the F0 and F1 generations, and the transgenerational phenotype in F2 (for review, see Bale, 2011; Gabory et al., 2009). Hence, more research is necessary to fully understand the role DNA methylation plays in the development of, or vulnerability for, PTSD. In addition to these issues, there are several confounds that must be considered in the human research that question the role of epigenetics in the transmission of stress effects across generations. For instance, it may be that the children of survivors of trauma adopt ineffective coping techniques like their parents. Alternatively, the children may have shared trauma with their parents, and/or been exposed to parental PTSD symptoms, or experienced parental emotional abuse or neglect. These, and others not listed, may contribute to the increased likelihood of a child developing a stress-induced psychopathology (Yehuda et al., 2005; Sturge-Apple et al., 2012; Lehrner et al., 2014). Both ethical limitations and the logistical constraints associated with human research limit full understanding of the mechanisms underlying these transgenerational effects. For this reason, recent efforts have been made to assess transgenerational effects of stress in rodents. Rodent models are useful because they: (A) simulate a human condition in a controlled setting; (B) allow the disease to be studied as it develops; and (C) facilitate the preliminary evaluation of pharmacological and other treatments for humans.

## Predator Stress as an Animal Model of PTSD

While there is no one ideal animal model of PTSD that recapitulates all symptoms of the disorder, Predator Stress paradigms are arguably one of the more comprehensive approaches used by researchers (see Deslauriers et al., 2017). Predator stress typically involves acute exposure of a prey species (mouse or rat) to a predator or predator cue (typically a cat, rat, or ferret). A considerable volume of literature exists demonstrating that cat exposure generates high levels of anxiety-like behavior, avoidance of trauma-related cues, hyperarousal, and impaired spatial memories (Adamec and Shallow, 1993; Adamec, 1998; Adamec et al., 1999, 2004, 2005, 2008; Blundell et al., 2005; Diamond et al., 2006; Cohen et al., 2009; Lebow et al., 2012; Fifield et al., 2013, 2015; Goswami et al., 2013; Zoladz et al., 2015; Lau et al., 2016; Schöner et al., 2017; Cohen et al., 2018). Other similarities to PTSD include the fact that female mice appear more susceptible to predator stress (Adamec et al., 2006) and common pharmacological treatments of PTSD (e.g., sertraline) are efficacious in reducing anxiety-like behaviors and hyperarousal following predator stress (Adamec et al., 2004; Matar et al., 2006). In addition, predator stressed rodents show the modifications in glucocorticoid levels, gene expression, and the release of CRH in the amygdala similar to that seen in PTSD (Adamec and Shallow, 1993; Adamec et al., 1998; Blanchard et al., 2001; Dielenberg et al., 2001; Cook, 2002; Hebb et al., 2003; Rosen, 2004; Schulkin et al., 2005; Takahashi et al., 2005; Roseboom et al., 2007; Armario et al., 2008; Campeau et al., 2008; Yao et al., 2008; Zoladz et al., 2008; Clinchy et al., 2011; Whitaker and Gilpin, 2015; Lau et al., 2016). Furthermore, predator stress induces long lasting potentiation of rodent amygdala afferent and efferent transmission in right hemisphere, the degree of which is highly predictive of changes in rodent anxiety (Adamec et al., 2005). Finally, amygdala dendritic length is increased following predator stress (Adamec et al., 2012; Cohen et al., 2014).

## Rodent Studies: Predator Stress Effects Across Generations

While predator exposure alters behavior indicative of stress-induced psychopathology in humans (see section above), there is growing laboratory evidence that predator stress during pregnancy has deleterious outcomes to rodent offspring. In mice, exposure to predator rat urine during the 1st week of pregnancy leads to a decrease in the litter size and survival of offspring (de Catanzaro, 1988). Predator odor exposure also induces heightened levels of circulating corticosterone in pregnant female rodents and alters offspring development (Weinstock et al., 1988). In rats, exposure to a live predator prenatally leads to a predisposition to seizures associated with alterations in hippocampal plasticity in the offspring (Ahmadzadeh et al., 2011; Saboory et al., 2011; Korgan et al., 2014). More recently, adult offspring (postnatal day 90) of mice exposed to a predator odor during the last half of pregnancy display increased predator-avoidance behavior, alterations in social behavior, novelty-induced anxiety-like behaviors, and increased corticosterone levels (St-Cyr and McGowan, 2015; St-Cyr et al., 2017, 2018). This is consistent with studies showing dexamethasone (synthetic glucocorticoid) administration during pregnancy leads to reduced HPA axis sensitivity in adult offspring by attenuating the GR and mineralocorticoid receptors in the hippocampus, which results in enhanced anxiety-like behaviors and stress responsivity (Levitt et al., 1996; Welberg and Seckl, 2001). While these data suggest that predator stress in the parental generation impacts offspring, much more research is required to fully elucidate these effects.

### Epigenetic Modifications: DNA Methylation and Predator Stress

As can be seen in Table 2, several studies have identified alterations in methylation states of specific genes following predator stress. For instance, Dlgap2, a gene that encodes a postsynaptic density protein, is more likely to be unmethylated in animals that display an anxious phenotype following the predator stress paradigm (Chertkow-Deutsher et al., 2010). Similarly, predator stress induces phenotypic variability in stress coping responses that can be linked to the degree of methylation of the hormone vasopressin (Avp) in the amygdala (Bowen et al., 2014). However, it is currently unknown whether changes in Dlgap2 and Avp DNA methylation persist into future generations.

**Table 2:** Selective rodent studies supporting the role of DNA methylation in predator stress model.

Reference	Candidate gene	Epigenetic changes	Sample size	Generation study carried out through
Chertkow-Deutsher et al. (2010)	*Dlgap2*	Higher *Dlgap2* methylation and reduced mRNA expression in predator odor exposed rat’s hippocampus.	Not mentioned	F0
Bowen et al. (2014)	*Avp*	Predatory stress was associated with decreased* Avp* promoter methylation in the medial amygdala	48	F0
St-Cyr and McGowan (2015)	*Bdnf*	Female offspring of mice exposed to predator odor during pregnancy had decreased *Bdnf* transcript abundance positively correlated with a concomitant decrease in methylation of *Bdnf* exon IV in the hippocampus	42	F0, F1(Intergeneration)
St-Cyr et al. (2017)	*Nr3c1, Fkfbp5*	Female offspring from prenatal predator odor-exposed dams showed increased transcript abundance of both the glucocorticoid receptor gene (*Nr3c1*; on the day of birth) and *Fkfbp5* (in adulthood) in the amygdala	24	F0, F1(Intergeneration)

*Abbreviations: amplification of inter-methylated sites (*Aims*), arginine vasopressin (*Avp*), brain-derived neurotrophic factor (*Bdnf*), disks large-associated protein 2 (*Dlgap2*), FK506 binding protein 5 (*Fkbp5*), nuclear receptor subfamily 3 group C member 1 gene (*Nr3c1*, glucocorticoid receptor gene)*.

Similar to human literature described above, methylation of the GR and Fkbp5 appear to play a role in the transmission of predator stress effects to future generations (see Table 2).

Female offspring from prenatal predator odor-exposed dams showed increased transcript abundance of both the GR gene (Nr3c1; on the day of birth) and Fkbp5 (in adulthood) in the amygdala (St-Cyr et al., 2017). Moreover, increased Fkbp5 expression was inversely correlated with decreased DNA methylation for this product’s gene (St-Cyr et al., 2017), a finding consistent with the human literature (Yehuda et al., 2016). In a related study, female offspring of mice exposed to predator odor during pregnancy had decreased *Bdnf* transcript abundance which was positively correlated with a concomitant decrease in DNA methylation of *Bdnf* exon IV in the hippocampus (St-Cyr and McGowan, 2015). Epigenetic alterations of the *Bdnf* gene have been linked to impaired brain functioning, memory, stress, and neuropsychiatric disorders (Fuchikami et al., 2010; Ikegame et al., 2013; Andero et al., 2014). These results are consistent with other work in which predator scent stress induced a significant down-regulation of *Bdnf* mRNA in the CA1 region of the hippocampus (Kozlovsky et al., 2007). Hypermethylation of hippocampal *Bdnf* DNA may be a cellular mechanism underlying the persistent hippocampus-specific cognitive deficits which are prominent features of the pathophysiology of PTSD. Indeed, selective hypermethylation of the *Bdnf* gene in the dorsal hippocampus appears to be an important component of local synaptic structure, plasticity, and maintenance of intrusive memories of the trauma in an active state following exposure to a life-threatening event (Zoladz et al., 2012). Despite these findings, more research is necessary to fully assess the role of DNA methylation in the transmission of stress effects across generations (Blouin et al., 2016). Overall, laboratory predator stress controlled experiments such as cross-fostering, *in vitro* fertilization, or experiments across multiple generations will provide invaluable information on the biological epigenetic transmission and behavioral transmission which is virtually impossible to achieve for ethical and practical reason in humans.

## Conclusion

Both human and rodent research suggests that stress effects can be passed on to future generations. However, there is a paucity of research on the mechanism of such a transmission. One possibility is epigenetic modifications. While the mechanism by which epigenetic modifications lead to the transmission of stress to subsequent generations is unknown, one possibility involves the transmission of DNA methylation. In humans, altered methylation of the GR, and/or its co-chaperone, FKBP5 genes, have been identified in the offspring of people with PTSD (Yehuda et al., 2004, 2014, 2015, 2016; Erhardt and Spoormaker, 2013). This is consistent with laboratory studies in rodents in which predator exposure during pregnancy alters methylation of the GR and FKBP5 DNA in offspring (St-Cyr et al., 2017). Future research is needed in both humans and animal models, however, to fully understand the role that DNA methylation (of specific targets such as GR) plays in the transmission of stress. Understanding mechanisms that promote PTSD will represent a major advance in the field and may lead to novel treatments for such a devastating, and often treatment-resistant disorder.

## Author Contributions

SB, AF, and JB wrote the article. PM and JD edited it.

## Conflict of Interest Statement

The authors declare that the research was conducted in the absence of any commercial or financial relationships that could be construed as a potential conflict of interest.

## Abbreviations

PTSD: post-traumatic stress disorder
OCD: obsessive compulsive disorder
GAD: generalized anxiety disorder
GR: glucocorticoid receptor
hGR: human glucocorticoid receptor
Fkbp5: FK506 binding protein 5
Bdnf: brain-derived neurotrophic factor
Dlgap2: Dlg associated protein 2
Avp: vasopressin
PND: postnatal day
Barx1: BARX homeobox
AIMS: Amplification of inter-methylated sites
ADCYAP1R1: ADCYAP receptor Type I
CDV: Community and domestic violence
CFTR: Cystic fibrosis transmembrane conductance regulator
COMT: Catechol-O-methyltransferase gene
CORIN: Corin, serine peptidase genes
IGF2: Insulin-like growth factor 2
IL8: Interleukin 8
IL16: Interleukin 16
IL18: Interleukin 18
Nr3c1: Nuclear receptor subfamily 3 group C member 1
Nr3c2: Nuclear receptor subfamily 3 group C member 2
SMYD3: SET And MYND domain containing 3
SLC6A4: Solute carrier family 6 member 4
qPCR: Quantitative PCR.
